# SDG2-Mediated H3K4 Methylation Is Required for Proper *Arabidopsis* Root Growth and Development

**DOI:** 10.1371/journal.pone.0056537

**Published:** 2013-02-19

**Authors:** Xiaozhen Yao, Haiyang Feng, Yu Yu, Aiwu Dong, Wen-Hui Shen

**Affiliations:** 1 State Key Laboratory of Genetic Engineering, International Associated Laboratory of CNRS-Fudan-HUNAU on Plant Epigenome Research, School of Life Sciences, Fudan University, Shanghai, PR China; 2 Institut de Biologie Moléculaire des Plantes du CNRS, Université de Strasbourg, Strasbourg Cedex, France; Temasek Life Sciences Laboratory & National University of Singapore, Singapore

## Abstract

Trithorax group (TrxG) proteins are evolutionarily conserved in eukaryotes and play critical roles in transcriptional activation via deposition of histone H3 lysine 4 trimethylation (H3K4me3) in chromatin. Several *Arabidopsis* TrxG members have been characterized, and among them SET DOMAIN GROUP 2 (SDG2) has been shown to be necessary for global genome-wide H3K4me3 deposition. Although pleiotropic phenotypes have been uncovered in the *sdg2* mutants, *SDG2* function in the regulation of stem cell activity has remained largely unclear. Here, we investigate the *sdg2* mutant root phenotype and demonstrate that *SDG2* is required for primary root stem cell niche (SCN) maintenance as well as for lateral root SCN establishment. Loss of SDG2 results in drastically reduced H3K4me3 levels in root SCN and differentiated cells and causes the loss of auxin gradient maximum in the root quiescent centre. Elevated DNA damage is detected in the *sdg2* mutant, suggesting that impaired genome integrity may also have challenged the stem cell activity. Genetic interaction analysis reveals that *SDG2* and *CHROMATIN ASSEMBLY FACTOR-1* act synergistically in root SCN and genome integrity maintenance but not in telomere length maintenance. We conclude that SDG2-mediated H3K4me3 plays a distinctive role in the regulation of chromatin structure and genome integrity, which are key features in pluripotency of stem cells and crucial for root growth and development.

## Introduction

During multicellular organism development, each cell type elaborates a specific developmental program, and the acquired cell fate needs to be stably maintained. The root is an important organ required for plant nutrients and water acquisition from the soil. The well-defined and rather stereotypical cell organization of *Arabidopsis* roots makes it an excellent experimental system to study cell fate maintenance and cell differentiation [1], [2]. The root meristem contains four types of stem cells: epidermis/lateral root cap initials, cortex/endodermis initials, stele initials, and columella root cap initials. These stem cells surround the quiescent centre (QC), which is composed of a small number of mitotically less-active cells, together forming the root stem cell niche (SCN). Each type of stem cell undergoes an asymmetric division to give rise to one daughter cell that maintains the stem cell status and the other daughter cell developing into a specific cell type. As such, a root is viewed as a bundle of cell files in which cells are aligned along an age gradient from the initial daughter cells to mature cells at the distal end. QC promotes the continuous cell division of the initial cells and provides short-range signals to prevent stem cells from differentiation [3].

Both hormone signaling and transcriptional networks regulate root growth and development. The phytohormone auxin is involved in almost all processes of root development including SCN formation [4]–[6], root elongation [7], lateral root (LR) positioning and development [8]–[10]. INDOLE-3-ACETIC ACID/AUXIN (IAA/Aux) proteins act as repressors of auxin-responsive transcription [11]. Gain-of-function of IAA family members blocks auxin-induced pericycle cell divisions for LR initiation and also results in other auxin-related phenotypes, including primary root growth arrest, limited root hair formation and reduced root gravitropism [9], [12]–[18]. In addition to auxin, other phytohormones such as cytokinins and brassinosteroids (BRs) are also involved in the regulation of root meristem activity [2]. Recent studies have shown that BRs act on the root meristem size control independently of auxin [19], [20].

The generation of a differentiated cell from a stem cell involves chromatin-based epigenetic reprogramming of the genome to establish the appropriate cell-specific transcription program. Several studies have shown that hair cells and non-hair cells at the *Arabidopsis* root epidermis differ in chromatin organization and histone modifications [21]–[23]. The evolutionary conserved histone chaperones, i.e. CHROMATIN ASSEMBLY FACTOR-1 (CAF-1), the NAP1-family proteins NRP1 and NRP2, and the ASF1-family proteins AtASF1A and AtASF1B, have been shown to be required for normal root growth [24]–[26]. Histone acetylations play important roles in both root epidermis patterning and SCN maintenance [27], [28]. SCN maintenance also requires appropriate Polycomb-mediated histone H3 lysine 27 (H3K27) methylation; while increased H3K27 trimethylation (H3K27me3) levels inhibit meristematic activity and root growth, reduced H3K27me3 levels enhance meristematic activity and root growth [29].

The evolutionary conserved Trithorax group (TrxG) proteins antagonize Polycomb group (PcG) proteins, together forming central regulators of cell identity that act by maintaining a tight balance between cell proliferation and cell differentiation [30]–[32]. Several *Arabidopsis SET DOMAIN GROUP* (*SDG*) genes have been identified and shown to exhibit TrxG-like H3K4-methyltransferase activity (reviewed in [33]). *ATX1*/*SDG27* regulates floral organ development through activating the expression of several homeotic genes [34]. *ATX1*/*SDG27*, *ATX2/SDG30* and *ATXR7/SDG25* all are involved in the activation of *FLOWERING LOCUS C* (*FLC*) expression and flowering suppression [35]–[38]. *ATXR3/SDG2* has a more prominent/pronounced role in H3K4me3 deposition and knockdown of its function leads to pleiotropic plant phenotypes including dwarfism, impaired male and female gametophyte development [39], [40]. In spite of these uncovered important roles of TrxG genes in plant growth and development, their functions in stem cell activity and cell fate determinacy remains to be explored.

In this study, we show that the loss-of-function mutant *sdg2-3* exhibits root growth arrest and produces fewer LRs as compared to wild-type (WT) plants. The postembryonic root growth defects in *sdg2-3* are caused by disorganization and meristem activity arrest of SCN in the primary roots and by suppression of SCN formation in LR development. Auxin signaling is partially perturbed in *sdg2-3* and exogenous application of auxin or BR cannot fully rescue the *sdg2-3* mutant root growth phenotype. In line with SDG2 function as a H3K4-methyltransferase, the H3K4me3 level in *sdg2-3* is reduced in root cells and in particular also in SCN cells. Genetic interaction analysis shows that *SDG2* and *CAF-1* synergistically regulate root growth and genome integrity maintenance. Our study thus highlights a distinct role of *SDG2* in regulation of genome function and root meristem activity.

## Results

### Loss of *SDG2* Impairs Root Growth and Development

**Figure 1:**
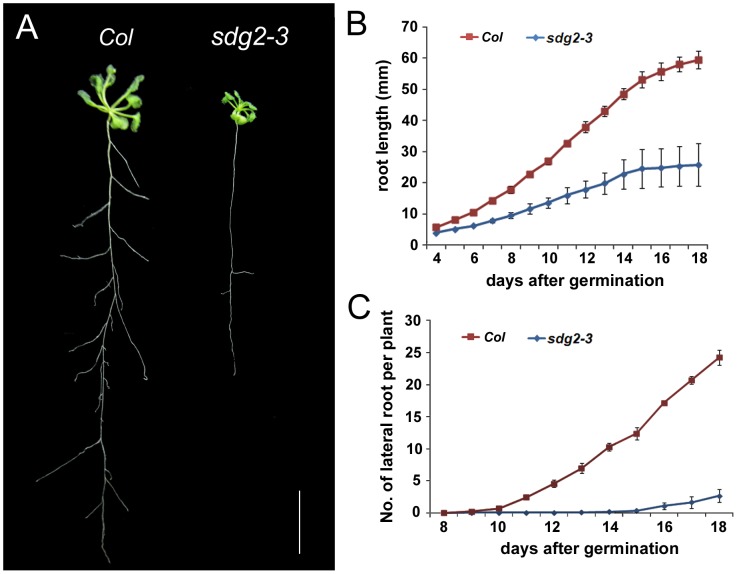
Loss of SDG2 reduces both primary and lateral root growth. A, Phenotypes of wild-type *Col* and the mutant *sdg2-3* seedlings at 26 days after germination. Bar = 1 cm. **B**, Comparison of primary root length between *Col* and *sdg2-3* from 4 to 18 days after germination. **C**, Comparison of lateral root number between *Col* and *sdg2-3* from 8 to 18 days after germination. All data are mean values from two independent experiments with each of at least 20 plants. Bars indicate SD.

Investigation of plant growth revealed that the examined allelic mutants *sdg2-1*, *sdg2-2* and *sdg2-3* [39] all exhibit a short-root phenotype (shown for *sdg2-3* in Figure 1A). Subsequently we focused on *sdg2-3* for more detailed analyses. Starting from the fifth day after germination the *sdg2-3* mutant compared to WT showed clear primary root growth retardation, and the difference became increasingly evident along with plant age, e.g the *sdg2-3* primary roots reached only about 50% mean length compared to those of WT in 18-day-old plants (Figure 1B). LR formation was also affected in *sdg2-3*. To the naked eye, WT seedlings produced the first LR at about 9 days of age whereas *sdg2-3* seedlings started only after 15 days. The LR number per plant is significantly lower in the mutant than in WT as evidenced from above 10-day-old seedlings (Figure 1C).

### *SDG2* is Required for Root SCN Organization and Function

**Figure 2:**
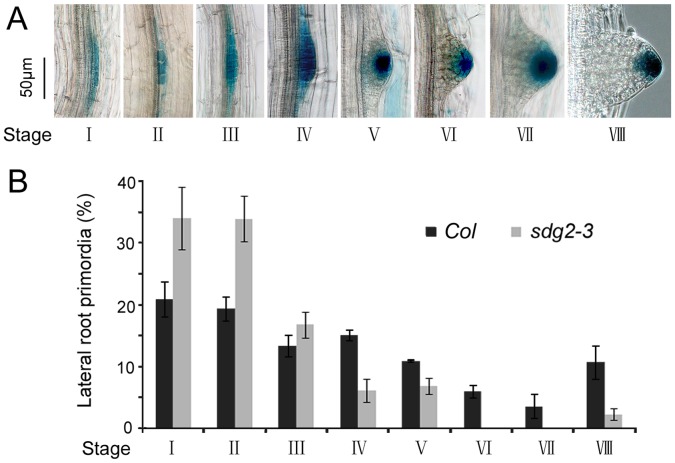
Loss of SDG2 distinctively affects different developmental stages of lateral root formation. A, Developmental stages of lateral root formation. Images was captured after histochemical GUS staining of roots from 10-day-old *Col* seedlings expressing *DR5:GUS.* Developmental stage nomenclature was according to Malamy and Benfey [43]. Bar = 50 µm. **B**, Relative distribution of developmental stages of lateral root primordia observed in 10-day-old seedlings of the wild-type *Col* and the mutant *sdg2-3*. Primordia were counted and examined for developmental stages from at least 20 plants, and the experiments were repeated three times. Mean values of percentage are shown and bars indicate SD.

The reporter line *DR5:GUS* marks the earliest events associated with LR formation [41], [42]. To investigate *SDG2* effects on LR formation in more detail, we introgressed *DR5:GUS* into *sdg2-3* by genetic crosses. Histochemical analysis revealed that *DR5:GUS* is expressed in all stages and morphologically recognizable LR primordia in *sdg2-3* as in WT (Figure 2A, developmental stage according to Malamy and Benfey [43]). Remarkably, we found that the majority of LR primordia from 10-day-old seedlings accumulate at developmental stage I and II in *sdg2-3* as compared to WT (Figure 2B). After stage IV LR development was drastically blocked in *sdg2-3* (Figure 2B). These observations support our previous idea and further provide detailed information that LR formation in *sdg2-3* is primarily inhibited from developmental stage VI on, at a time when a critical mass of cells is reached to form a structured root SCN.

**Figure 3:**
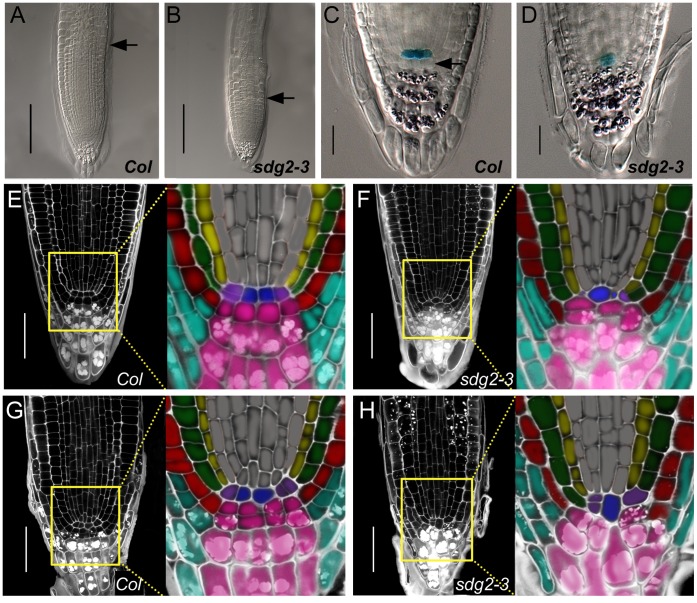
Loss of SDG2 impairs the primary root stem cell niche maintenance. A and **B**, Comparison of primary root apical meristem sizes between wild-type *Col* and the mutant *sdg2-3*, respectively. DIC images were taken on the roots of 6-day-old seedlings. Arrowheads indicate positions of the transition from meristem to elongation zone. Bar = 100µm. **C** and **D**, Comparison of *QC25:GUS* expression and root cap cell layer organization between *Col* and *sdg2-3*, respectively. DIC images were taken on GUS- and Lugol-stained root tips of 6-day-old seedlings. Arrowheads indicate the columella initial cell layer. Bar = 20 µm. **E** and **F**, Comparison of cell layer organization of root apical meristem between *Col* and *sdg2-3*, respectively. Confocal images were taken on PI-stained roots of 6-day-old seedlings. Bar = 50 µm. The close-up regions are shown by color indication of different cell types: QC cell in blue, columella root cap and columella initial cells in rose, lateral root cap cells in sky-blue, epidermal cells and epidermis/lateral root cap initials in red, cortex cells in green, endodermal cells in yellow, cortex/endodermis initials in purple, stele cells and stele initials in gray. **G** and **H**, Comparison of cell layer organizations of root apical meristem between *Col* and *sdg2-3*, respectively. Confocal images were taken on PI-stained roots of 14-day-old seedlings. Bar = 50 µm. The close-up regions are shown with colorations as described in **E** and **F**.

We further examined SCN organization in the primary roots. Consistent with the short-root phenotype, the size of the root apical meristem (RAM) was reduced in *sdg2-3* compared to WT (Figure 3A and 3B). A close examination of the root tip revealed that WT roots contain the regular and arc-shaped arrangement of the four layers of starch granule-rich columella cells and a layer of starch granule-lacking columella initial cells located under the QC layer (Figure 3C). In *sdg2-3* roots, the columella cells were displayed in disorganized cell layers and starch granules were observed in cells adjacent to QC (Figure 3D), indicating a loss of columella initial cell identity. In addition, expression of the QC specific marker *QC25:GUS* [44] was detected at lower levels and in a fewer number of cells in *sdg2-3* compared to WT roots (Figure 3C, D). Further propidium iodide (PI) staining and microscopy analysis revealed that, compared with WT (Figure 3E), the *sdg2-3* mutant contains a disorganized SCN with reduced number of QC cells, fewer and less recognizable stem cells of stele initials, as well as fewer cortex/endodermis initials, epidermis initials, and columella root cap initials (Figure 3F). The cell size was also largely more variable, with either increased or reduced volume, in each type of cells within SCN of *sdg2-3* as compared to WT. As compared to the so far described defects in the primary roots of 6-day-old *sdg2-3* seedlings, the primary roots of 14-day-old *sdg2-3* seedlings showed similar SCN defects but to a more severe degree (Figure 3H), whereas the regular SCN organization in WT was stably maintained (Figure 3G). It appears that during postembryonic seedling growth the *sdg2-3* root SCN gradually loses cell identity and stem cell function, causing root growth arrest.

Taken together, our results indicate that *SDG2* function is required for SCN establishment for LR development and is critical for stable maintenance of SCN organization and function in primary roots.

### Auxin Regulation is Partly Disrupted by Loss of *SDG2* Function

**Figure 4:**
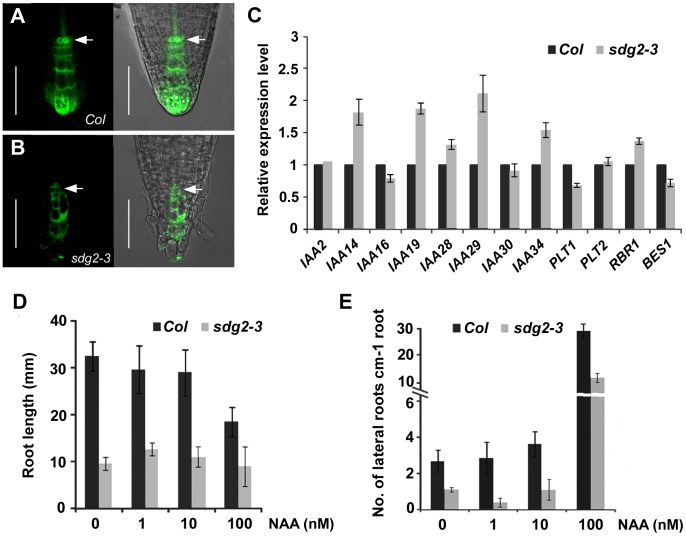
Loss of SDG2 partially affects auxin regulation in roots. A and **B**, Comparison of the expression pattern of *DR5:GFP* reporter in 5-day-old wild-type *Col* and the mutant *sdg2-3*, respectively. Note that auxin gradient maximum in QC visualized by *DR5:GFP* expression in *Col* is lost in *sdg2-3*. Bar = 50 µm. **C**, Relative gene expression levels determined by quantitative RT-PCR analysis. RNA was prepared from roots of 20-day-old *Col* or *sdg2-3* seedlings. RT-PCR was performed using gene specific primers and normalized using *ACTIN2* as reference. Relative expression levels of the indicated genes are shown as mean values from three biological repeats and with *Col* value setting as 1. Bars indicate SD. **D**, Effects of exogenous NAA on root elongation of *Col* and *sdg2-3* seedlings. Seeds were germinated and grown on medium containing the indicated concentration of NAA. Root length is shown as a mean value obtained from three independent experiments with each experiment comprising 20 plants. Bar indicates SD. **E**, Effects of exogenous NAA on lateral root (LR) formation of *Col* and *sdg2-3* seedlings. LR and primordia were counted using the GUS reporter of 10-day-old *Col* or *sdg2-3* seedlings expressing *CYCB1;1:GUS*. The total number of LR and primordia was divided by root length to report LR formation ability of individual plants. Mean values obtained from three independent experiments and 20 plants per sample per experiment are shown, and bars indicate SD.

To gain further insight into the mechanisms underlying the *sdg2-3* root SCN defect, we introgressed into *sdg2-3* the marker *DR5:GFP* which reports auxin signaling in single cells [45], [46]. In WT roots expressing *DR5:GFP*, the GFP signal was detected at high levels in columella cells, columella initial cells and QC cells (Figure 4A). In *sdg2-3* roots, the intensity of GFP signal appeared slightly weaker and most importantly the auxin gradient and maximum in QC were lost; almost no GFP signal could be detected at QC position (Figure 4B). Next, we performed quantitative real-time RT-PCR analysis for auxin-related genes to compare their expression in WT and *sdg2-3* roots. As shown in Figure 4C, expression of each *IAA14*, *IAA19, IAA29, IAA34*, and to a lesser extent of *IAA28*, was significantly increased whereas expression of *IAA2, IAA16* and *IAA30* was unchanged in *sdg2-3*. An increase of expression in *sdg2-3* was also observed for the cell cycle inhibitory gene *RETINOBLASTOMA RELATED* (*RBR*, Figure 4C). Because *RBR* and several *IAA* genes are known to negatively regulate root growth [9], [14], [17], [47], their upregulation is consistent with the root growth suppression phenotype of the *sdg2-3* mutant. Nevertheless, because *SDG2* acts as an activator of gene transcription [39], [40], it is likely that the observed gene upregulation is caused indirectly by the *sdg2-3* mutation. An auxin-mediated *PLETHORA* (*PLT*) pathway is essential for root SCN maintenance [48], consistently the expression of *PLT1* (but not *PLT2*) was reduced in *sdg2-3* roots (Figure 4C).

We addressed the question as to whether auxin supply would rescue the *sdg2-3* mutant phenotype. Root growth was investigated in the presence of various concentrations of exogenous 1-naphthalene acetic acid (NAA). We found that root growth is less responsive to NAA inhibition in *sdg2-3* compared to WT (Figure 4D). Nevertheless, in no case *sdg2-3* root growth could reach that of WT. To examine LR development in the presence of NAA, we introgressed into *sdg2-3* the marker *CYCB1;1:GUS*, which reports cell division activity and lateral root primordia formation [49], [50] (Supplementary Figure S1). At low concentrations (1 or 10 nM), an NAA effect on LR formation was not evident. However, NAA at 100 nM drastically stimulated LR formation in WT and *sdg2-3* (Figure S1 and 4E). In the latter case, the LR and primordia number per root length was still significantly lower in *sdg2-3* compared to WT (Figure 4E). Taken together, our data indicate that loss of *SDG2* function affects partially auxin regulation but the mutant plants remain responsive to auxin and exogenous auxin supply could not fully rescue the mutant root defects. A slight downregulation of *BES1*, which encodes a key transcription factor of the BR signaling pathway [51], was observed in *sdg2-3* (Figure 4C). However, similar to auxin exogenous brassinolide (BL, a type of bioactive BR) also could not fully rescue the *sdg2-3* mutant root defects (Supplementary Figure S2). It appears that *SDG2* determines root meristem activity not only through a specific phytohormone-signaling pathway.

### *SDG2* and *CAF-1* Synergistically Regulate Root Meristem Activity

**Figure 5:**
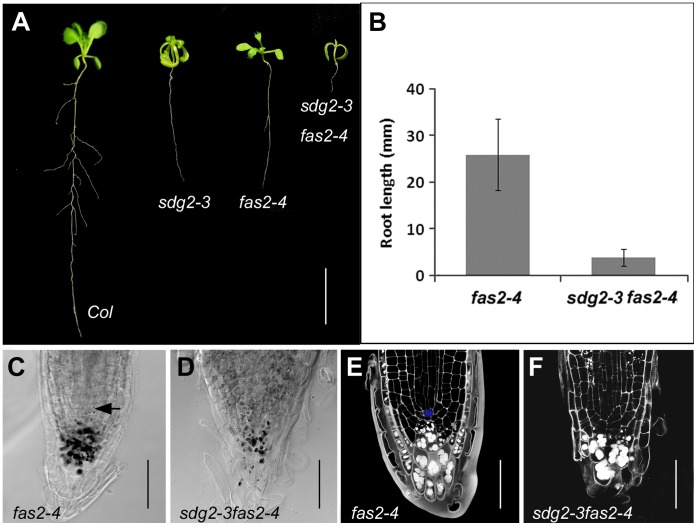
Loss of SDG2 synergistically enhances growth defects of the CAF1 loss-of-function mutant *fas2-4*. A , Representative example of 14-day-old seedling of the wild-type *Col*, the single mutants *sdg2-3* and *fas2-4,* and the double mutant *sdg2-3 fas2-4*. Bar = 1 cm. **B**, Comparison of primary root length between *fas2-4* and *sdg2-3 fas2-4* on 16-day-old seedlings. Root length is shown as a mean value from two independent experiments with each comprising at least 20 plants. Bar indicates SD. **C** and **D**, Comparison of root cap cell organization between *fas2-4* and *sdg2-3 fas2-4*, respectively. DIC images were taken on Lugol-stained root tips of 6-day-old seedlings. Arrowhead in **C** indicates QC position. Bar = 50 µm. **E** and **F**, Comparison of cell layer organizations of root apical meristem between *fas2-4* and *sdg2-3 fas2-4*, respectively. Confocal images were taken on PI-stained roots of 6-day-old seedlings. The QC cell in **E** is marked in blue. Bar = 50 µm.

CAF-1 regulates histone deposition in chromatin and the loss-of-CAF-1 mutants *fas1* and *fas2* exhibit multiple defects of root development, including loss of SCN [24], perturbed cell fate at epidermis [21], and compromised LR development [52]. We asked whether SDG2 and CAF-1 act in a same regulatory pathway. To address this question, we generated the *sdg2-3 fas2-4* double mutant by genetic crosses between the *sdg2-3* and *fas2-4* single mutants. The double mutant showed a drastically arrested growth phenotype (Figure 5A). While the *sdg2-3* and *fas2-4* single mutants showed a similar short-root phenotype, a synergistic effect of *sdg2-3* and *fas2-4* on root growth inhibition was observed (Figure 5A and 5B). The mean root length of the double mutant *sdg2-3 fas2-4* reached to less than 20% of that of *fas2-4* at 16 days after germination (Figure 5B). The regular arrangement of the cell layers at RAM was disturbed in *fas2-4* (Figure 5C and 5E), which is in agreement with the previous report on another mutant allele *fas2-1* [24]. Compared with the single mutant *fas2-4* (Figure 5C), the double mutant *sdg2-3 fas2-4* showed much fewer starch granules at the root tip cells (Figure 5D). The PI staining and microscopy analysis showed that the typical cellular organization of SCN was totally lost in *sdg2-3 fas2-4* (Figure 5F). The severe disorganization and loss of SCN are consistent with the drastic root growth defects observed in *sdg2-3 fas2-4*. The synergistic effect of *sdg2-3* and *fas2-4* indicates that *SDG2* and *CAF-1* act in genetically parallel pathways and they are independently required for the maintenance of root SCN organization and stem cell activity.

### *SDG2* is Involved in Global H3K4me3 Deposition in Root Cells and in the Maintenance of Genome Integrity

**Figure 6:**
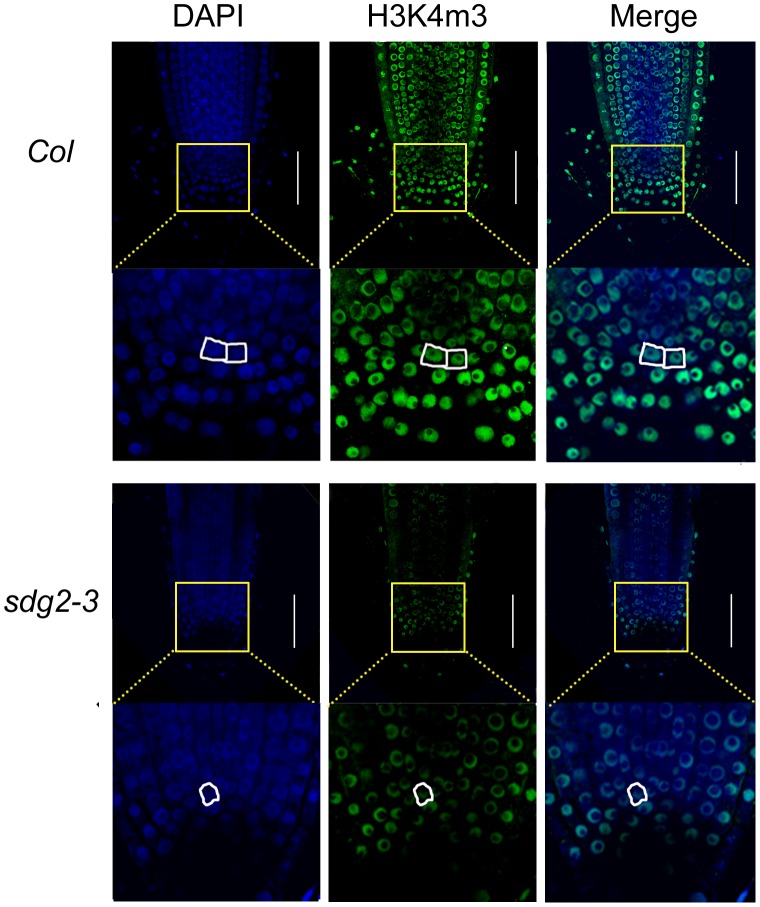
Loss of SDG2 drastically reduces nuclear H3K4me3 levels in root cells. Whole-mount root immunofluorescence staining was performed using an antibody specifically recognizing H3K4me3. Panels from left to right subsequently show confocal images of DAPI, H3K4me3 and merged signals. Close-up images show regions around the root stem cell niche with the QC cells circled. Bar = 50 µm.

Finally, we addressed the questions whether *SDG2* is required for H3K4me3 deposition in specific root cells and whether it affects genome integrity. Previous western blot analyses have shown that H3K4m3 level is reduced in *sdg2* mutant plants [39], [40]. We analyzed H3K4me3 levels in individual cells by whole-mount root immunofluorescence [53]. In WT roots, a strong H3K4me3 signal was detected in the nuclei of all cells except for stele cells which showed a weak H3K4me3 signal (Figure 6). In *sdg2-3* roots, the H3K4me3 signal was drastically reduced, with only a small number of cells showing clear visible immunofluorescence (Figure 6). Most remarkably, root SCN cells and in particular QC cells showed strong H3K4me3 immunostaining in WT but a very weak signal in *sdg2-3* (Figure 6). These observations demonstrate that SDG2 is a H3K4-methyltransferase required for global H3K4me3 deposition in root cells, and that impaired H3K4me3 deposition correlates with interrupted stem cell function in the *sdg2-3* mutant root SCN.

**Figure 7:**
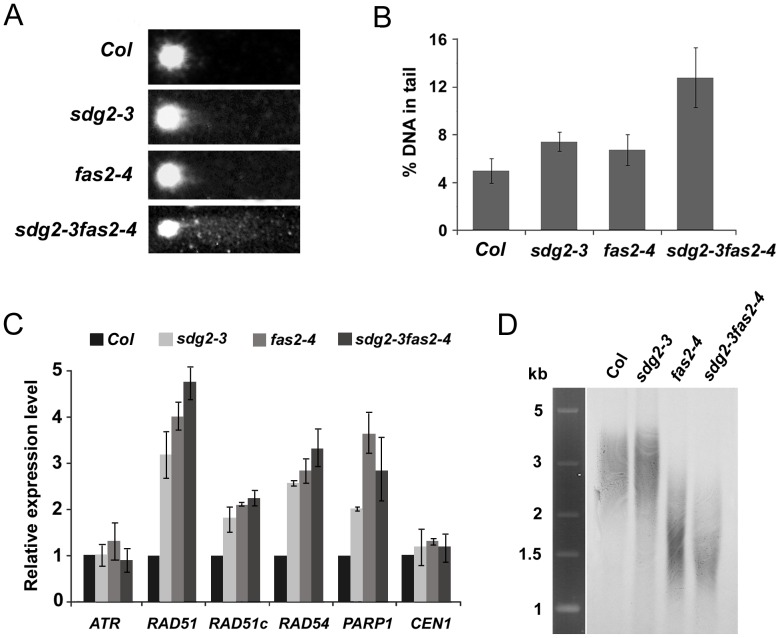
Loss of SDG2 synergistically enhances the CAF1 loss-of-function mutant *fas2-4* in causing genome DNA damage but not telomere shortening. **A,** Representative comet images of the wild-type *Col* and the mutants *sdg2-3*, *fas2-4* and *sdg2-3 fas2-4*. Note the intact nucleus at the left and comet tail formed by fragmented nuclear DNA to the right on each panel. **B,** DNA damage levels as measured by the percentage of DNA in the comet tails of nuclei for the wild-type and mutants. The mean value of more than 100 nuclei is shown with a SD bar. **C,** Relative expression levels of DNA repair genes determined by quantitative RT-PCR analysis. RNA was prepared from 14-day-old *Col*, *sdg2-3*, *fas2-4* or *sdg2-3 fas2-4* seedlings. RT-PCR was performed using gene-specific primers and normalized using *ACTIN2* as reference. Relative expression levels of the indicated genes are shown as mean values from three biological repeats and with *Col* value setting as 1. Bars indicate SD. **D,** Telomere length comparison between wild-type and mutants. Genomic DNA was digested with *Mse*I, and DNA gel blot analysis was performed using a DIG-labeled telomere repeat as the probe. Note that telomeres are shortened to similar degree in *fas2-4* and *sdg2-3 fas2-4* but not in *sdg2-3* as compared to *Col*.

Dynamic chromatin and stringent protection of genome integrity are important features of plant SCN. Indeed, histone chaperone (CAF-1, NRPs or ASF1) mutants exhibiting root growth defects also show increased DNA damage [25], [26], [54], [55]. We performed comet assay to investigate the level of DNA damage in WT and mutants *sdg2-3*, *fas2-4* and *sdg2-3 fas2-4* seedlings. Figure 7A shows typical nuclei comets observed in WT and mutants. As shown in Figure 7B, the percentage of DNA in comet tails was slightly increased in *sdg2-3* and *fas2-4* seedlings compared to that of WT. In the *sdg2-3 fas2-4* double mutant, the DNA damage level was drastically enhanced (Figure 7B), indicating a synergistic role of *SDG2* and *CAF-1* in the protection of genome integrity. Consistent with increased levels of DNA damage, several genes involved in DNA repair (including *RAD51*, *RAD51c*, *RAD54* and *PARP1*) were activated in the mutants (Figure 7C). The *sdg2-3 fas2-4* double mutant behaved relatively similar to the single mutants in DNA repair gene activation (Figure 7C), indicating that the observed gene activation is not quantitatively correlated with DNA damage levels. Finally, we investigated telomere length in WT, *sdg2-3*, *fas2-4* and *sdg2-3 fas2-4.* Consistent with previous reports [55], [56], *fas2-4* caused telomere shortening (Figure 7D). In contrast, *sdg2-3* had no detectable effect on telomere length (Figure 7D). These results indicate that SDG2 differs from CAF-1 in regulating genome integrity and chromatin function.

## Discussion

In this study, we provide evidence that TrxG gene and H3K4me3 play crucial roles in root stem cell fate establishment and maintenance during plant postembryonic development. We have demonstrated that: 1) the TrxG-family member SDG2 is necessary for H3K4me3 deposition in root cells, particularly in the SCN cells; 2) the loss-of-function mutant *sdg2-3* exhibits SCN disorganization and stem cell termination, causing primary root growth arrest; 3) *sdg2-3* also exhibits defects in SCN establishment, causing inhibition of LR development. Our finding reinforces the current thought that chromatin structure is crucial in pluripotency, which is a defining feature of stem cells, and that reprogramming of chromatin epigenetic states occurs and accompanies cell differentiation and cell fate establishment throughout plant ontogeny.

Previous studies showed that SDG2 specifically methylates H3K4 *in vitro* and loss of SDG2 results in global genome-wide reduction of H3K4me3 *in planta* [39], [40]. The *SDG2* gene is ubiquitously expressed in various plant organs including roots, stems, leaves, inflorescences, young floral organs and primordia [39], [40]. Consistently, the loss of SDG2 mutants display pleiotropic phenotypes including shorter roots, smaller rosettes, shorter stems and impaired gametophyte development. The transition from vegetative to reproductive growth has been reported to be altered in *sdg2* mutants in some studied conditions but not always consistently [39], [40], [57]. The precise cause of discrepancy remains unknown. The severely affected growth in *sdg2* mutants likely makes flowering more influenced by environmental conditions, and also introduces a layer of complexity in flowering time measurement (by days to flowering or by leaf number at flowering). Our study, here focused on roots, discovers a critical function of SDG2-mediated H3K4me3 in SCN establishment and maintenance. Distinct from the loss of CAF-1 mutants (*fas1* or *fas2*) that show root growth inhibition but shoot fasciation [24], the *sdg2* mutants show both root and shoot growth inhibition. Strikingly, the double mutant *sdg2-3 fas2-4* shows drastically enhanced defects of root SCN organization and function, and also of shoot growth (Figure 5A). Future studies will be required to investigate shoot SCN to uncover the cellular and molecular mechanisms underlying common and specific roles of the chromatin regulators SDG2 and CAF-1 in the regulation of root and shoot stem cell activity.

We have shown that the auxin gradient maximum which appeared in the QC cells in WT was lost in *sdg2-3* roots (Figure 4). Previous genetic analysis shows that auxin acts upstream of the major regulators of stem cell activity [4], [6], and the QC ablation experiment demonstrates that reestablishment of auxin maximum is earlier than the re-specification of a new QC in root [46]. We thus believe that the loss of auxin accumulation and gradient is a potent cause of the irregular cell shape and position of QC in *sdg2-3*. High levels of auxin promote the proteosome-mediated degradation of IAA proteins, which act as repressors of auxin response by binding Auxin Response Factors (ARFs) to regulate downstream gene transcription. Several IAA genes, including the previously characterized ones: *IAA14*, *IAA19* and *IAA28* [9], [14], [17], are upregulated in *sdg2-3*, further supporting a perturbed auxin pathway by loss of SDG2 function. Nonetheless, *sdg2-3* is still responsive to auxin in root elongation and LR formation tests, and its defective root growth/developmental phenotype cannot be fully rescued to WT in all our studied growth conditions.

Another cause of the mutant root growth/developmental defects might reside in the altered transcriptional reprogramming of the genome associated with H3K4me3 reduction in chromatin. Transcriptome analysis on 15-day-old (accession GSE39898) or 10-day-old (accession GSE23208 [40]) seedlings has revealed that about 10% of all genes are misregulated in the *sdg2* mutant. Many genes encoding transcription factors are among the downregulation category of misregulated genes. It is currently unknown whether any of these genes constitute ‘master regulators’ of root growth and/or development. Loss of CAF-1 can alter H3.1 and H3.3 composition within *Arabidopsis* chromatin [52] and differences exist between the H3.1 and H3.3 methylations [58], pointing to a possible mechanism of crosstalk between SDG2 and CAF-1. Yet, the number of misregulated genes is much lower in *fas2* seedlings (<1% of all genes [59]), and there is no overlap of genes misregulated in *sdg2* and *fas2*. The synergistic effects of *sdg2-3* and *fas2-4* on plant growth and development also highlight an independent function between *SDG2* and *CAF-1*. It is likely that *SDG2* and *CAF-1* regulate independent but interactive pathways to determine stem cell activity.

Genome stability is also affected in *sdg2-3*. Loss of SDG2 leads to slightly but significantly increased levels of DNA damage in plants grown under normal growth conditions. DNA damage is drastically enhanced in the *sdg2-3 fas2-4* double mutant. Interestingly, in contrast to the telomere shortening by *fas2-4*, *sdg2-3* does not affect telomere length, indicating that DNA damage and telomere length maintenance are unlinked. H3K4 methylation also plays an important function in DNA repair in other organisms. In budding yeast, the H3K4-methyltransferase Set1 is recruited to newly induced double strand break (DSB) sites and induces *de novo* H3K4me3 deposition on the nucleosomes around the DSB site [60]. The *set1* mutant displays reduced capacity to repair DSB by non-homologous end joining [60]. In human cells, H3K4me3 is reduced to an almost undetectable level at DSB sites and an accumulation of the H3K4me3-demethylase JARID1A has been observed at damaged regions [61]-[63]. The mechanisms causing the elevated levels of DNA damage in *sdg2-3* remain currently unknown. Our analysis does not support that a reduced transcription of DNA repair genes causes elevated DNA damage. On the contrary, several DNA repair genes are expressed at higher levels in *sdg2-3* compared to WT (Figure 7C). Regardless of the mechanism involved, elevated DNA damage can seriously challenge stem cell activity. Root and shoot stem cells are particularly sensitive to DSBs caused by physical or chemical agents [64]. Stringent protection of genome integrity is necessary for stem cells to guarantee genetic inheritance and identity in daughter cell populations. Future studies will investigate the mechanisms linking chromatin dynamics and genome integrity maintenance to further appreciate these important features of pluripotency of stem cells.

TrxG and PcG play antagonistic roles in the regulation of cell fate maintenance in animals and plants. In plants, this has been demonstrated primarily in *Arabidopsis* flowering time control and floral organ identity determinacy. In roots, loss of function of the PcG-family member CLF causes increased root length, increased RAM and stem cell activity [29]. These are opposite to the root defects caused by loss of SDG2 function, suggesting that PcG and TrxG also antagonistically regulate root stem cell activity. Further knowledge about the mechanisms of PcG and TrxG function will be essential to understand how cellular pluripotency and cell differentiation have evolved on the way to multicellularity in plants and in animals.

## Materials and Methods

### Plant Materials and Growth Conditions

*Arabidopsis* ecotypes used in this study are from the Columbia accession. The mutant *sdg2-3* and *fas2-4* have been described [39], [55], and the reporter lines *QC25:GUS*, *CYCB1;1:GUS*, *DR5:GUS* and *DR5:GFP* have also been described previously [41], [44], [45], [49]. Higher order combinations of mutants were produced in this study by genetic crosses.

For *in vitro* plant growth, seeds were surface sterilized (70% ethanol and 0.1% Tween 20 for 10 min) and then plated on square plates containing agar Murashige and Skoog medium (MS salts, 1% sucrose, pH 5.8, 0.8% bacto-agar). For phytohormone treatment, the agar MS medium contains the specified concentrations of NAA or BL. The plates were cold-treated for 2 days at 4°C to synchronize germination. Plates were then incubated in a nearly vertical position in a growth cabinet at 21°C under a 16h light/8h dark photoperiod.

### Phenotypic Analysis

For growth comparison, WT and mutant plants were grown side by side on a same plate. Primary root length was determined by measuring from root tip to hypocotyl base. Emerged LR was counted by naked eye observation. All data are mean values of at least 20 plants, and the experiments were repeated twice.

### GUS Assays

GUS activity of WT or mutant seedlings containing *QC25:GUS, DR5:GUS* or *CYCB1;1:GUS* was assayed by incubating whole seedlings in a staining solution comprising 0.04% 5-bromo-4-chloro-3-indolyl-β-d-glucuronide, 50 mM NaHPO_4_ pH 7.0, 2 mM K_4_Fe(CN)_6_, 2 mM K_3_Fe(CN)_6_, 5 mM EDTA, 0.1% Triton X-100 for 3–6 hours at 37°C. Roots were mounted and imaged using a Imager A2 microscope (Zeiss, http://microscopy.zeiss.com). GUS stained LR and primordia were counted from at least 20 plants, and the experiments were repeated three times.

### Dye Staining and Confocal Microscopy

For starch granule staining, roots were immersed for 3 to 5 min in Lugol iodine solution containing 5% iodine and 10% potassium iodide, rinsed with water, cleared with chloral hydrate solution (chloral hydrate: water: glycerol, 8∶3:1, w:v:v). Differential interference contrast (DIC) images were acquired with a Imager A2 microscope (Zeiss). PI staining was performed as previously described [65]. Confocal image analysis was performed using a LSM710 microscope (Zeiss). The excitation wavelength for PI fluorescence was 488 nm and the emitted fluorescence was detected through a 520–721 nm band pass filter. GFP fluorescence imaging of living cells from WT or mutant roots expressing *DR5:GFP* was performed using excitation wavelength at 488 nm and a 493–598 nm band pass filter.

### Gene Expression Analysis

Total RNA was isolated using the TRlzol kit according to standard procedures (Invitrogen, http://www.invitrogen.com). Reverse transcription was performed using Improm-II reverse transcriptase (Promega, http://www.promega.com). Quantitative RT-PCR was performed as described [66]. In all experiments, three biological replicates of each sample and three technical (PCR) replicates were performed. *ACTIN2* was used as a reference gene to normalize the data. The gene specific primers used are listed in Supplemental Table S1.

### Immunostaining and H3K4me3 Detection

Immunofluorescence staining was performed according to the previously described method [53]. Briefly, 4-day-old seedlings were fixed and treated with Driselase (Sigma, http://www.sigmaaldrich.com). After washing, the samples were incubated with the anti-trimethyl-H3K4 antibody (Upstate Catalogue no. 07–473, http://www.millipore.com). Alexa Fluor 488-conjugated anti-rabbit IgG antibodies (Invitrogen, A11008) were used as the second antibody. Imaging was performed using the LSM710 confocal microscope (Zeiss). The H3K4me3 signal was detected using the excitation wavelength at 488 nm and a 493–598 nm band pass filter. The DAPI (4′,6-diamidino-2-phenylindole) staining signal was detected using the excitation wavelength at 405 nm and a 410–585 nm band pass filter. All staining, imaging and processing conditions are strictly the same for the wild-type *Col* and the mutant *sdg2-3*.

### Comet Assay and Telomere Length Analysis

14-day-old seedlings were used for comet assay. Comet assay and the following evaluation were performed as described [25]. Images of comets were captured under the Imager A2 microscope (Zeiss). The comet analysis was performed using CometScore software (http://autocomet.com). 4-week-old seedlings were used in telomere length analysis according to the previously described protocol [55], [56].

### Accession Numbers

SDG2– At4g15180, IAA2– At3g23030, IAA14– At4g14550, IAA16– At3g04730, IAA19– At3g15540, IAA28– At5g25890, IAA29– At4g32280, IAA30– At3g62100, IAA34– At1g15050, PLT1– At3g20840, PLT2– At1g51190, RBR1– At3g12280, BES1– At1g19350, ACTIN2– At3g18780, FAS2 – At5g64630, ATR – At5g40820, RAD51 – At5g20850, RAD51c – At2g45280, RAD54 – At3g19210, PARP1 – At4g02390, CEN1– At3g50360.

## Supporting Information

Figure S1 **Histochemical GUS staining patterns of** ***CYCB1;1::GUS*** **in NAA treatments experiments.** The well-characterized marker line *CYCB1;1::GUS* indicate lateral root and lateral root primordia by marking active cell division. 10-day-old *CYCB1;1::GUS*/*Col* and *CYCB1;1::GUS/sdg2-3* seedlings grown on MS medium or MS medium supplemented with 100 nm NAA were collected for histochemical GUS staining. 10-day-old *sdg2-3* produce much less lateral root compared with *Col*. 100 nM NAA treatment drastically induce the lateral root formation in both *Col* and *sdg2-3,* however, the increased number of the LR and primordia was still significantly lower in *sdg2-3* compared to *Col*. Bars = 2 mm.
(TIF)

Figure S2 **Brassinosteroid sensitivity of** ***Col*** **and** ***sdg2-3*** **roots. A**, Effects of exogenous brassinolide (BL) on root elongation of *Col* and *sdg2-3* seedlings. Seeds were germinated and grown on medium containing the indicated concentration of BL. Root length is shown as a mean value obtained from three independent experiments and each experiment of 20 plants. Bar indicates for SD. Application of BL from 1 nm to 100 nm can inhibit the root elongation of *Col* plants. In *sdg2-3* plants, this inhibition of root growth was not very significant, **B**, Effects of exogenous BL on lateral root (LR) formation of *Col* and *sdg2-3* seedlings. LR and primordia were counted using the GUS reporter of 10-day-old *Col* or *sdg2-3* seedlings expressing *CYCB1;1::GUS*. The total number of LR and primordia was divided by root length to report LR formation ability of individual plant. Mean values obtained from three independent experiments and 20 plants per sample per experiment are shown, and bars indicate for SD. Application of BL stimulated lateral root formation in both *Col* and *sdg2-3* plants. However, in all of the BL concentration we tested, the LR and primordia number per root length was still significantly lower in *sdg2-3* compared to *Col*. Our data indicate exogenous BL supply could not fully rescue the mutant root defects.
(TIF)

Table S1 (DOC)
