# Supplementary figures and images for: SDG2-Mediated H3K4 Methylation Is Required for Proper Arabidopsis Root Growth and Development
Source: PLoS One. 2013 Feb 19;8(2):e56537. doi: 10.1371/journal.pone.0056537 (PMC3585709; doi:10.1371/journal.pone.0056537)

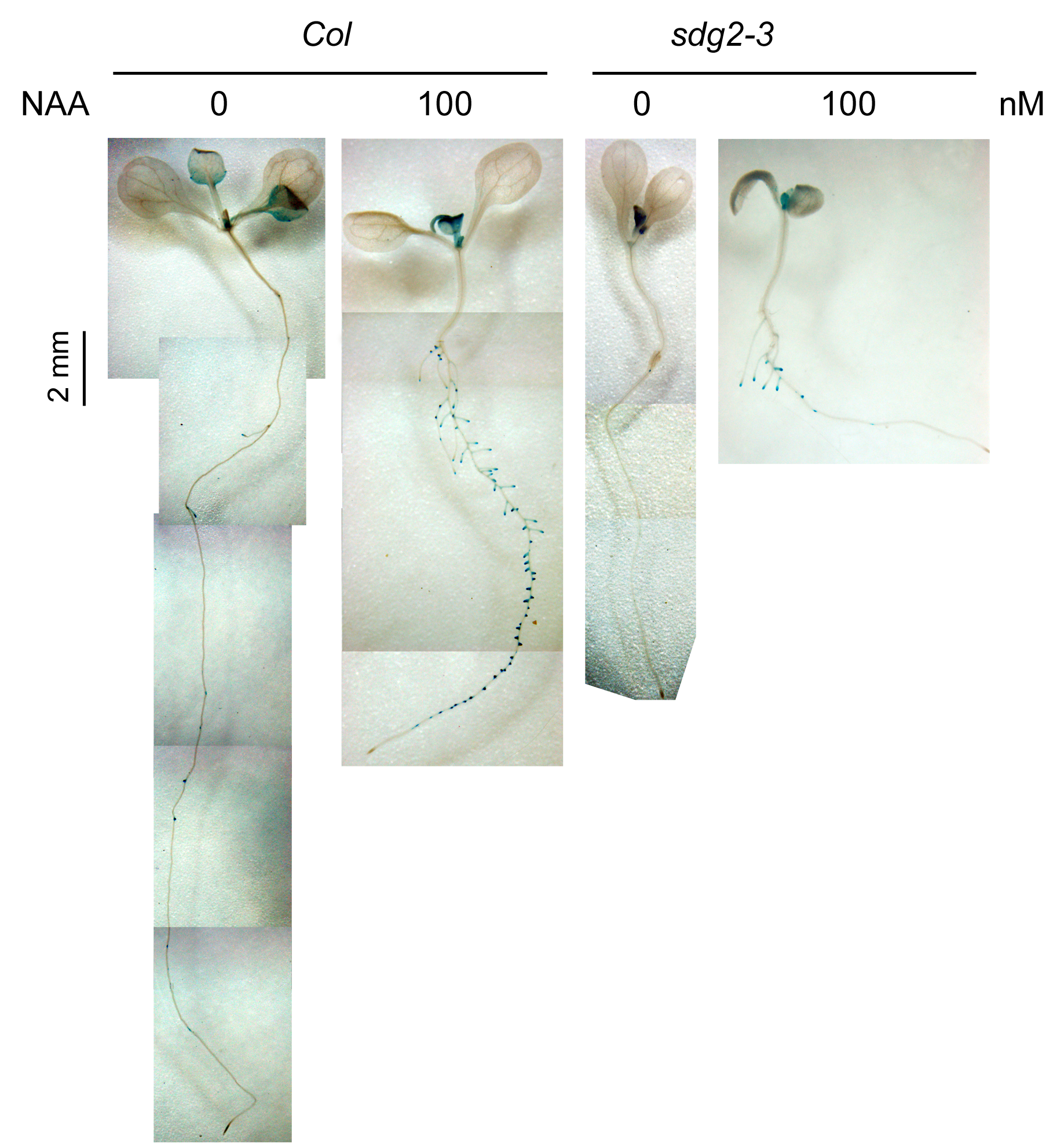

Supplement: Figure S1 — Histochemical GUS staining patterns of CYCB1;1::GUS in NAA treatments experiments. The well-characterized marker line CYCB1;1::GUS indicate lateral root and lateral root primordia by marking active cell division. 10-day-old CYCB1;1::GUS/Col and CYCB1;1::GUS/sdg2-3 seedlings grown on MS medium or MS medium supplemented with 100 nm NAA were collected for histochemical GUS staining. 10-day-old sdg2-3 produce much less lateral root compared with Col. 100 nM NAA treatment drastically induce the lateral root formation in both Col and sdg2-3, however, the increased number of the LR and primordia was still significantly lower in sdg2-3 compared to Col. Bars = 2 mm. (TIF) [file pone.0056537.s001.tif]

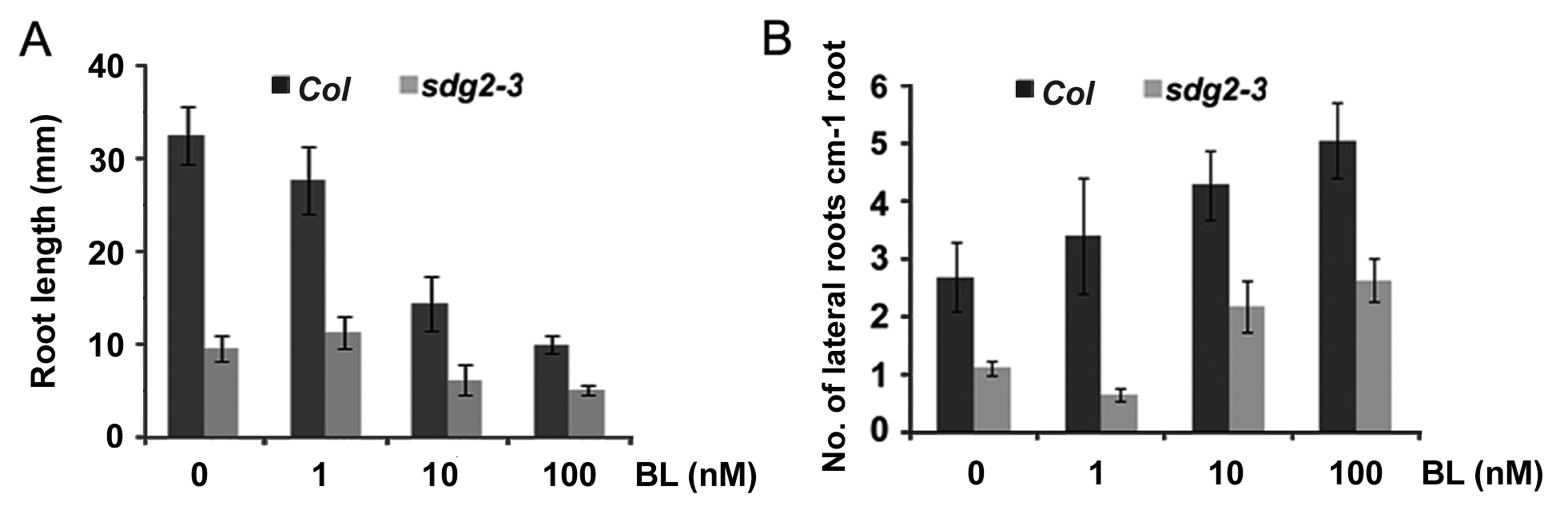

Supplement: Figure S2 — Brassinosteroid sensitivity of Col and sdg2-3 roots. A, Effects of exogenous brassinolide (BL) on root elongation of Col and sdg2-3 seedlings. Seeds were germinated and grown on medium containing the indicated concentration of BL. Root length is shown as a mean value obtained from three independent experiments and each experiment of 20 plants. Bar indicates for SD. Application of BL from 1 nm to 100 nm can inhibit the root elongation of Col plants. In sdg2-3 plants, this inhibition of root growth was not very significant, B, Effects of exogenous BL on lateral root (LR) formation of Col and sdg2-3 seedlings. LR and primordia were counted using the GUS reporter of 10-day-old Col or sdg2-3 seedlings expressing CYCB1;1::GUS. The total number of LR and primordia was divided by root length to report LR formation ability of individual plant. Mean values obtained from three independent experiments and 20 plants per sample per experiment are shown, and bars indicate for SD. Application of BL stimulated lateral root formation in both Col and sdg2-3 plants. However, in all of the BL concentration we tested, the LR and primordia number per root length was still significantly lower in sdg2-3 compared to Col. Our data indicate exogenous BL supply could not fully rescue the mutant root defects. (TIF) [file pone.0056537.s002.tif]
