# Supplementary material for: SDG2-Mediated H3K4 Methylation Is Required for Proper Arabidopsis Root Growth and Development
Source: PLoS One. 2013 Feb 19;8(2):e56537. doi: 10.1371/journal.pone.0056537 (PMC3585709; doi:10.1371/journal.pone.0056537)
Supplement: Table S1 — (DOC) [file pone.0056537.s003.doc]

**Table S1.** RT-PCR primers used in this study.

| Primers used in RT-PCR | | |
| --- | --- | --- |
|  | Forward primer | Reverse primer |
| *IAA2* | CGTTGGTTGGCCACCAGTGA | ACGCTTTGAGAAGCTCGGGGT |
| *IAA14* | CAGCTCCTTTACCATGGGGAG | ACCAACGAGCATCCAGTCAC |
| *IAA16* | TGGGATGGCCACCGGTACGA | CACGGTGGCACATGCGGAGG |
| *IAA19* | CGTGGCATCGGTGTGGCCTT | GCTGCAGCCCAAACCCGGTA |
| *IAA28* | GCTCCTCCTTGTCACCAATTCACT | ACTGGAGCTACCTCAACCCTGTTA |
| *IAA29* | TGTGCGATCGAGGGTGCTGC | CGTCTTCCTCGTTGGGCTGGC |
| *IAA30* | GAGACTCGGGCTCAGCTTCGGA | CTCTGCCGCACCGACTCCAT |
| *IAA34* | GCAGCGATCCTCCCCATCCCT | ACGCCACCAAACTCCGTGGTC |
| *PLT1* | AAGCTTCACCATCCGAGACC | CCAGTCCATCGATGTCTTGTGA |
| *PLT2* | CAAGTGGAGGAGCCATCGTT | ACCGGTCCATCTATGTCTTGT |
| *RBR1* | AAAGCCGCTGCTGGTGGAGC | CACCTTTGGCGATCCGGGACA |
| *BES1* | CGCCAGTTCCATGCTCCGGC | GGTAGGCGAGGTTGGCACCAT |
| *ATR* | AACCCACATGCTCAGCGGGC | TCAACCGGCGAGCCTGACCT |
| *RAD51* | CGCCATTTCCCTCCACTCTCAAGC | ACCTGCTGCCTGAAGCTGTTCG |
| *RAD51c* | TGACACACCACGACACAGTCACA | TGGGCTTTATGAAACAATTGGGCCG |
| *RAD54* | TGAGAGACAGGTGGGCACTCC | ACGTCACCTCGTCACCTGCTGA |
| *PARP1* | TGCTCGCGCGAACTCACTTCT | AGCCTCTCCACCAGAACGGCT |
| *CEN1* | TCAACAAAATGATAGCTGATGTGGATA | CTGGAATGCTTTAGTGAGCTCTTCTT |
| *ACTIN2* | AAGTCATCACGATTGGTGCTG | ACCTGAGAACATAGTGGTTCC |
